# Breast Cancer Reporting in Lagos, Nigeria: Implications for Training and Education in Africa

**DOI:** 10.1200/JGO.2015.003079

**Published:** 2016-04-06

**Authors:** Adetola O. Daramola, Adekunbiola A. Banjo, Aneliese Bennett, Fatimah Abdulkareem, Abeer M. Shaaban

**Affiliations:** **Adetola O. Daramola**,** Adekunbiola A. Banjo**, and **Fatimah Abdulkareem**, Lagos University Teaching Hospital, Lagos, Nigeria; **Aneliese Bennett**, St James’s University Hospital, Leeds, United Kingdom; **Abeer M. Shaaban**, Queen Elizabeth Hospital, and the University of Birmingham, Birmingham, United Kingdom.

## Abstract

**Purpose:**

To assess the completeness and accuracy of breast cancer pathologic reporting in Nigeria.

**Materials and Methods:**

The histologic parameters provided in breast cancer pathology reports at a large teaching hospital in Nigeria were assessed. The corresponding slides were reviewed after the United Kingdom Royal College of Pathologists guidelines, and results were compared.

**Results:**

Out of 115 breast cancer cases, histologic type of breast carcinoma was concordant with the review type in 53.1% of cases and discordant in 46.9%. Grading was stated in 89.62% of cases, of which 50.5% were correctly graded, 35.8% were under-graded, and 8.5% were over-graded. Poor fixation and omission of the mitotic count were the main reasons for discordant grades. A comment on lymph node status was included in 40% of cases, and lymphovascular invasion was not commented on in 97.4% of cases. Only 26% of the tumors had hormone receptors and/or *HER2* tested.

**Conclusion:**

Some essential histologic parameters were absent from the histologic reports, and where present, a proportion were inaccurate. Attention to specimen fixation and method of grading and familiarity with uncommon breast cancer types are required; all can be facilitated by education and training. The use of a template/proforma is recommended to ensure cancer data set parameters are included in the pathology reports.

## INTRODUCTION

The most common presentation of lesions that affect the female breast in Nigeria is a symptomatic breast lump, and malignant tumors compose 20% to 26% of those lumps.^1^ There is an increasing incidence of breast cancer in African countries, including Nigeria;^2^ however, the actual burden of the disease is unknown. This is partly because there is no existing national program for early detection and management of the disease. Systematic collection of high-quality data, including vital parameters that help to inform patient management such as grade, histologic type, nodal status, and hormone receptor and human epidermal growth factor receptor (HER2) status, is required. Compilation of a complete data set nationwide that includes these parameters is currently impossible, because breast cancer reporting practice vary across hospitals. A guideline for the handling and reporting of breast samples produced by the Nigeria Breast Pathology Working Group^3^ in 2010 has yet to be circulated, implemented, and evaluated in most centers.

In Africa, only a few countries are known to have guidelines for reporting breast cancer,^3,4^ and it is not known from the available literature how well pathology reports conform to universally accepted guidelines for breast cancer reporting. At the Lagos University Teaching Hospital, a large tertiary referral center in Nigeria, attempts to standardize breast cancer reporting have been ongoing since 2005. Before this time, < 25% of breast carcinomas were graded; however, between 2007 and 2011 this increased to an average of 75% of cases,^1^ corresponding to an increased collaboration with colleagues in diaspora through various training and educational initiatives.

The aim of this study, therefore, was to determine the compliance and concordance of breast cancer histologic parameters reported at a large Nigerian laboratory with the cancer data set of the Royal College of Pathologists (RCPath), United Kingdom. This is to inform training and education requirements for African pathologists and ultimately improve patient management.

## MATERIALS AND METHODS

Breast cancer cases were identified from the pathology database of the Lagos University Teaching Hospital, Lagos, Nigeria. Data were extracted from histopathology reports covering a period from January 2011 to March 2013. Only reports of female patients with ductal carcinoma in situ (DCIS) and/or invasive carcinomas were included. Representative tumor slides and paraffin-embedded blocks were collected and re-embedded, and hematoxylin and eosin sections were prepared. All tumors were jointly reviewed by two pathologists (A.M.S., a United Kingdom specialist breast pathologist; and A.O.D., a Nigerian pathologist) after the RCPath guidelines^5^ to confirm diagnosis, type, grade, and nodal status. Patients’ ages and clinical data, where available, were collected from the original pathology reports. Both pathologists were blinded to the reported diagnoses. Histologic parameters on the original histology reports were compared with results of the histologic review. In addition, representative tumor areas were selected and marked for tissue microarray (TMA) construction.

### TMA Construction

A manual tissue microarrayer (MTA1; Beecher Instruments, Sun Prairie, WI) was used to construct the TMAs using 0.6-mm core punches, with a 1-mm interval between cores, as previously described.^6,7^ Two cores in duplicate were punched out from the marked areas of the donor blocks (center and periphery of tumor where possible) and arrayed into the prepunched holes in the recipient block according to the location on the TMA map. Of each TMA block, 3-µm sections were cut onto Superfrost slides and numbered sequentially for immunohistochemical staining.

### Immunohistochemistry

Standard immunohistochemistry was done using four specific monoclonal antibodies for estrogen receptor (ER; clone NCL-L-ER-6F11; dilution 1:250; Leica, Milton Keynes, United Kingdom), progesterone receptor (PR; clone PgR 636; dilution 1:800; Leica), HER2 (clone A0485CB11; dilution 3.6 µL:4 mL; Dako, Cambridgeshire, United Kingdom), and E-cadherin (clone M3612; dilution 1:100; Dako), as previously described.^6,8^ The secondary detection system was done using the Dako Envision Kit, and staining was done using the Dako Immunostainer 48. ER/PR/HER2 staining and scoring were done after the guidelines of the RCPath and the National External Quality Assessment Service as per the standard diagnostic protocols for the Leeds Teaching Hospitals National Health Service Trust.^5^ HER2 borderline 2+ cases were not subjected to further testing.

## RESULTS

A total of 115 breast carcinomas fulfilled the inclusion criteria and were reviewed. Table 1 summarizes the percentage of reports that stated the RCPath cancer data set parameters.

**Table 1:**
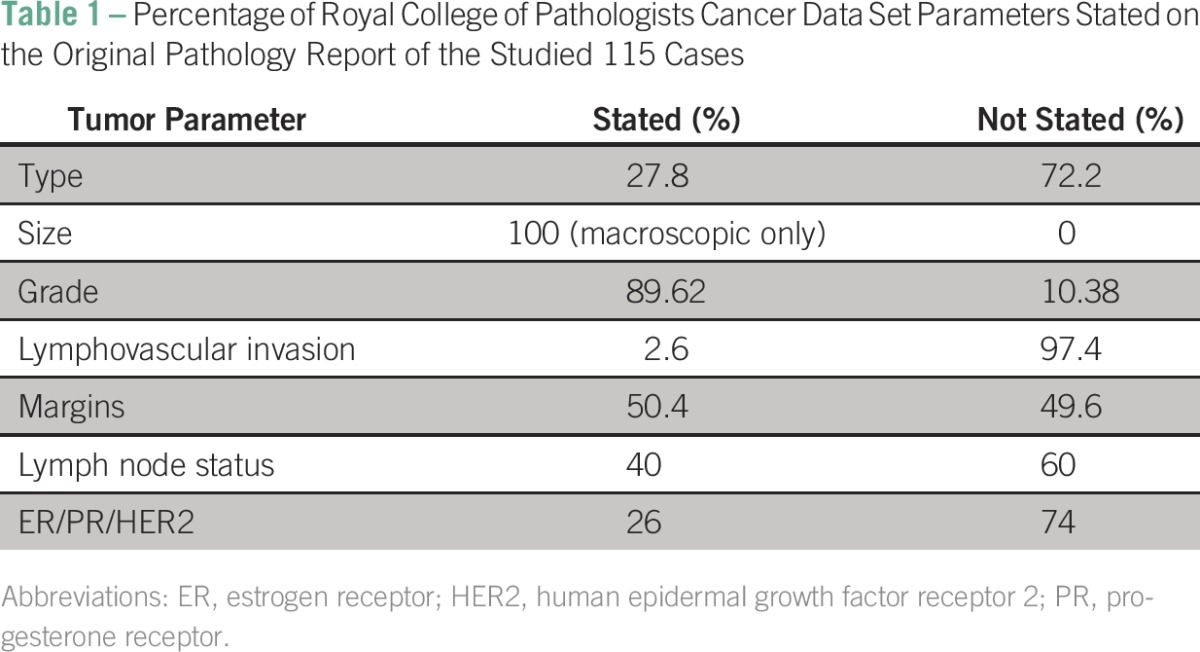
Percentage of Royal College of Pathologists Cancer Data Set Parameters Stated on the Original Pathology Report of the Studied 115 Cases

**Fig 1:**
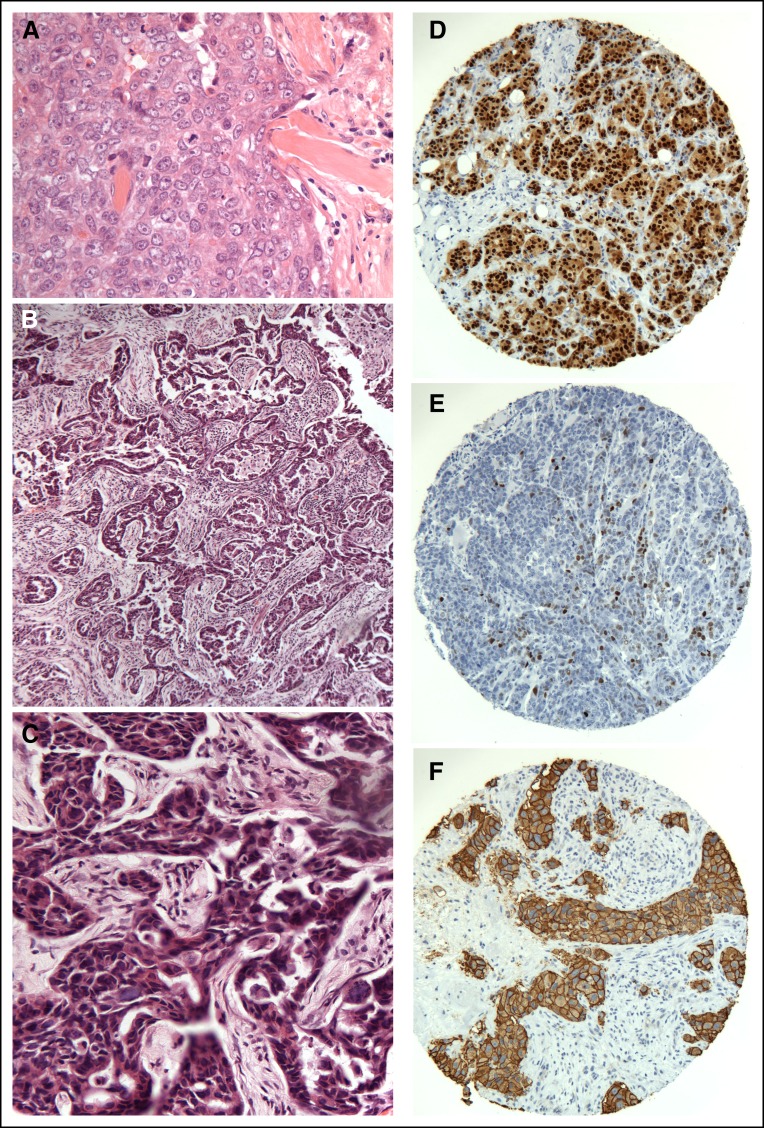
Histologic and immunohistochemical features of the Nigerian breast cancer. (A) A typical example of an invasive ductal carcinoma grade 3, showing syncytial growth pattern, marked nuclear pleomorphism, and frequent mitoses. (B) A low-power example of an under-graded invasive carcinoma (grade 1 on the original report) as a result of the prominent tubule formation. (C) A high-power view of the same tumor showing marked nuclear pleomorphism and conspicuous mitoses. This tumor was classed as grade 3 after histologic review. (D) Estrogen receptor strongly positive, (E) progesterone receptor patchy positivity, and (F) human epidermal growth factor receptor 2–positive invasive carcinomas in tissue microarrays.

The histologic type was stated in the pathology report in 27.8% of cases. Where present, this was concordant in 53.1% of cases and discordant in 46.9%. In 47% of cases, the histologic type was stated as invasive ductal carcinoma with no further qualification. On review, invasive carcinoma of no special type was the most common histologic type, accounting for 63.5% (73 of 115) of cases. Those tumors commonly showed diffuse pattern, necrosis, lymphocytic infiltrate, and conspicuous mitoses (Fig 1A). This was followed by the mixed type, accounting for 14.8% (17 of 115). DCIS was seen as the sole lesion in 7% (8 of 115) of cases. Concomitant DCIS was infrequently commented on. No cases of pure lobular carcinoma in situ or pleomorphic lobular carcinoma in situ were identified. The four cases of invasive pleomorphic lobular carcinoma were not recognized by the original reporting pathologist. E-cadherin was negative in all invasive lobular carcinoma cases identified. A high proportion of metaplastic carcinoma, 14.8% (17 of 115) was noted (Table 2).

**Table 2:**
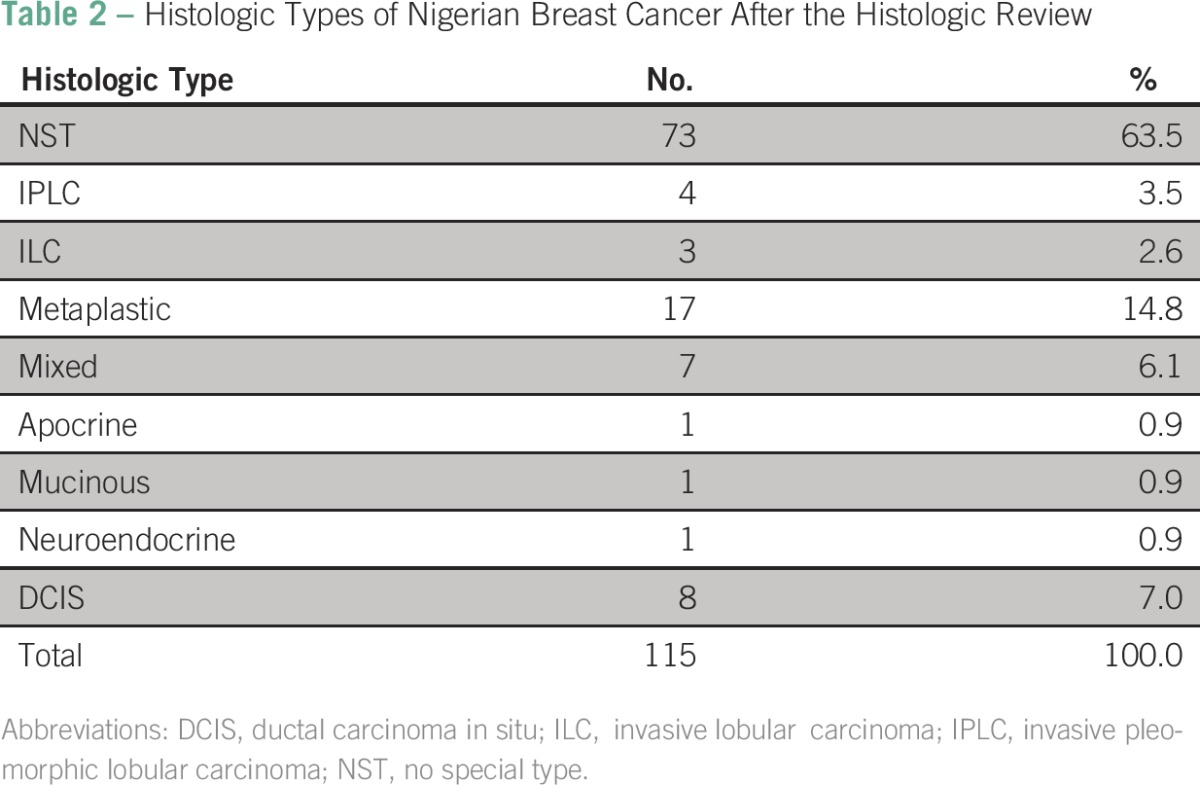
Histologic Types of Nigerian Breast Cancer After the Histologic Review

With respect to grading, 89.62% lesions were graded in the original report; 50.5% were correctly graded, 35.8% were downgraded, and 8.5% were upgraded. Detailed analysis showed that under-grading was due to omitting or under-scoring the mitotic count together with overemphasizing tubule formation (Figs 1B and 1C).

The macroscopic tumor size was available in all cases, but this was not confirmed or revised on the basis of the microscopic findings. No reports included a figure for the whole tumor size. A comment on lymph node status was available in 40% of cases.

On staining for hormone receptors and HER2, 26.9% of cases were ER positive (Fig 1D), 15.8% PR positive (Fig 1E), 6.1% HER2 score 3+ (Fig 1E), and 5.1% borderline 2+. Only 26% of the 115 cases reviewed had prior hormone receptor and HER2 testing done. This number was insufficient to adequately assess for concordance of marker expression.

## DISCUSSION

Breast cancer in Africa is an ever-increasing health issue, and accurate diagnosis and reporting are the first steps toward improved patient management. In Western countries, guidelines for standardized pathology reporting have been in use for decades. Only a few African countries, such as Nigeria and Uganda, are known to have published guidelines for reporting breast cancer,^3,4^ and the issue of lack of national guidelines has also been recently raised in other countries, including Ghana (A. Titloye, personal communication, December 2013). So far, no evaluation of the implementation of the available guidelines has been attempted in any African center. This article provides information, for the first time to our knowledge, on the adequacy of breast cancer reporting in Nigeria as compared with the cancer data set of the RCPath.

Grading was consistently reported in approximately 90% of reports, and this is a major advance. At the commencement of efforts to assess and include grade in Nigerian reports a few years back, only a minority of reports commented on the grade.^1^ The current study, however, reveals that further training in grading is required, because grade was accurately assessed in only half of the reports. The nodal status, which is recognized to be the most important prognostic parameter in breast carcinoma, was stated in less than half of the cases (40%). Other important histologic parameters, such as lymphovascular invasion and state of the relevant surgical margins, were often absent.

Under-grading, the major reason for discordant grading in this series, was predominantly due to omission/under-scoring of the mitotic count and relying on tubule formation as the main parameter for grading (Figs 1B and 1C). Poor fixation was evident in the majority of sections examined; this is a recognized cause for under-scoring the mitotic count, missing vascular invasion as well as under-scoring hormone receptors and HER2 immunohistochemistry.^9,10^ Since this study, protocols for adequate fixation have been implemented at the Lagos laboratory. In addition, training on the components of grading and importance of using tissue reference cells for comparison is being instituted by United Kingdom pathologists via a voluntary external quality assessment (EQA) scheme that runs twice a year in Nigeria.

The study also highlights the importance of recognizing which data should be included in the pathology report and how to accurately assess them, for example, recognizing special types of breast cancer and the role of immunohistochemistry (eg, E-cadherin) in confirming the diagnosis. In this context, the use of proforma would be valuable as aide-mémoire for the practicing pathologist.

The minimum data set that must appear on pathology reports to adequately plan therapy and determine prognosis includes the following vital parameters: size of tumor (in the largest dimension), distance between tumor and the nearest radial margin, histologic type of tumor, histologic grade, the presence or absence of vascular invasion, and nodal status.^5,11,12^ Our study showed that some of these important parameters were absent in many reports (Table 1). The histologic type, where stated, was accurate in 53.1%. Histologic type has been shown worldwide to be the most consistently reported element in breast cancer, with tumor size, grade, and margin reported with less consistency.^13-15^

The use of proformas is widely accepted as a practice that increases the adequacy of a pathology report. In a study by Idowu et al,^15^ 2,125 cancer reports were reviewed in 86 institutions across the United States; 31.2% of reports had some vital element(s) missing. Institutions in which checklists were routinely used reported a higher rate of complete reports than those who were not routinely using checklists (88% *v* 34%). Extent of invasion and status of resection margin were more likely to be missing in the incomplete reports. Mathers et al^16^ also reported a significant improvement in the completeness of histopathologic reporting in breast cancer cases after the introduction of a structured standard proforma. The use of proforma for cancer reporting should be encouraged in all African histopathology laboratories to ensure completeness of reports. The RCPath cancer data set proforma is available to download, free of charge, from the RCPath website.^17^

In Nigeria, efforts to standardize histopathology reports are ongoing. Standardization of breast cancer reporting, in particular, has been a priority since 2005 and received a boost in 2010 with the production of a booklet on minimum standards for breast cancer reporting.^3^ This was initiated by a Nigerian Breast Pathology Working Group formed at a workshop on quality assurance in breast pathology for pathologists and medical laboratory scientists from all over the country. Many centers, however, continue to use different methods of reporting because of lack of follow-up training. Diagnostic EQA schemes are also known to help with diagnostic quality assurance and education. There is no compulsory EQA scheme in Nigeria. However, since May 2011, a voluntary EQA scheme has been running regularly twice a year in the country.^18^ Slide sets (23 to 25 cases) are circulated throughout the country, with provision for a confidential online response via the EQA page of the course website. Working closely with Nigerian and United Kingdom counterparts as facilitators, regional EQA meetings are held to review the cases. Although there are challenges of running an EQA scheme without regulatory bodies, the educational aspect has positively affected both trainees and trainers alike.

Isolated efforts at quality assurance are helpful but cannot produce a sustainable improvement in diagnostic pathology services in the country. A type of regulatory support provided by the RCPath and UK National External Quality Assessment Service would be welcome. Structured support from willing professional bodies, such as the Association of Clinical Pathologists and International Academy of Pathology (IAP) and/or its divisions that can work with the local professional bodies, like the African Divisions of the IAP (eg, West African Division of the IAP and East African Division of the IAP) and African Colleges of Pathology, would help establish quality assurance processes in those African countries that require this type of support

In conclusion, vital histopathologic parameters, such as tumor type, grade, vascular invasion, and nodal status, can be missing or inaccurate within African histopathology reports. In recent years, despite infrastructural and socioeconomic challenges in Nigeria, moderate progress has been made toward the standardization of breast cancer reporting. This has largely been influenced by exposure to more training opportunities within and outside the country. A structured consistent approach to education, training, and quality assurance issues is needed to produce a sustained improvement in diagnostic pathology. The use of proformas, with the inclusion of all the main parameters, would ensure adequacy of reports.

## Appendix

Presented in part at the San Antonio Breast Cancer Symposium, San Antonio, TX, December 10-14, 2013; the Royal College of Pathologists, United Kingdom, Pathology is Global Symposium, London, United Kingdom, November 5, 2014; and the Royal College of Pathologists, United Kingdom, International Pathology Day, November 18, 2015, London, United Kingdom.
